# Data on the rheological behavior of cassava starch paste using different models

**DOI:** 10.1016/j.dib.2018.06.112

**Published:** 2018-06-30

**Authors:** Modupe Elizabeth Ojewumi, Kayode Gbolahan Oyeyemi, Moses Eterigho Emetere, Joshua Olusegun Okeniyi

**Affiliations:** Covenant University, P.M.B 1023, Km 10, Idiiroko. Canaan Land, Sango Ota, Ogun State, Nigeria

## Abstract

Proper selection of rheological models is very important in flow characterization. These models are often used to evaluate parameters that help in the characterization of food samples. Rheological models also provide flow predictions for extreme conditions where the flow nature of the fluid cannot be determined, hence the need for appropriate selection of rheological models. The principal aim of this study is to suggest a rheological model that best characterize the rheological behavior of native cassava starch and to determine the effect of state variables like temperature and concentration on the accuracy of rheological models. Five rheological models (i.e. Herschel-Bulkley model, Robertson-Stiff model, Power-law model, Bingham plastic model and Prandtl-Eyring model) were selected for this study and these models were modified into statistical models by the inclusion of the error variance (*ε*). The least-square method was used in evaluating the various model parameters for each model. From this study, it was seen that the Herschel-Bulkley model and the Robertson-Stiff model most accurately described the rheological patterns in cassava starch production. The sensitivity analysis of the different rheological models also shows that the accuracy of the Herschel-Bulkley model, Robertson-Stiff model and Power-law model is not significantly affected by variations in temperature and concentration of the cassava starch. However, it was observed that the Bingham plastic model and Prandtl-Eyring model gave less accurate predictions at higher concentration and lower temperature respectively. A lot of the industrially accepted models such as the Bingham plastic model may not necessarily be the best model for characterization cassava starch production as shown in this study, hence rheological model optimization is recommended for further study.

## Nomenclature

*A*: Model parameter for Roberston-Stiff, Prandtl-Eyring fluid model
*B*: Model parameter for Roberston-Stiff, Prandtl-Eyring fluid model
*f*(*γ*, *β*): General expression for rheological models
Matlab: Matrix Laboratory Software
*M*: Number of model parameters
*N*: Number of data points
*R*^2^: Coefficient of determination
RMS: Residual Mean Square in Ibf^2^/100ft^4^
RSS: Residual Sum of Square in Ibf^2^/100ft^4^
*µ*: value of model parameter
*ε*: Random error in Ibf/100 ft^2^
*τ*: Shear stress in Ibf/100 ft^2^
*τ*_0_: Yield stress in Ibf/100 ft^2^
*μ*_p_: Plastic viscosity, Ibf s/100 ft^2^
*γ*: Shear rate in s^−^^1^
*γ*_o_: Yield shear rate

**Specifications Table**

**Table:** 

Subject area	*Chemical Engineering*
More specific subject area	*Rheology*
Type of data	*Table, graph, figure*
How data was acquired	*Laboratory and Modelling*
Data format	*Raw, filtered and Analyzed data*
Experimental factors	*Statistical modelling was used.*
Experimental features	*Five rheological models (i.e. Herschel-Bulkley model, Robertson-Stiff model, Power-law model, Bingham plastic model and Prandtl-Eyring model) were selected for this study and these models were modified into statistical models by the inclusion of the error variance (ε).*
Data source location	*Ogun State, Nigeria*
Data accessibility	*Data set is with this article*

**Value of the data**

• The dataset will help to investigate the rheological properties of cassava starch.
• Data will assist in developing best model for cassava starch characterization using Statistical optimization of five rheological models.
• Effect of state variables like temperature and concentration on the accuracy of rheological models will be determined using the dataset.

### 1 Data

Dataset provided in this work revealed that investigations of rheological measurement does not only involve flow behaviour of liquids, but also on solids deformation behaviour. This research work examines the rheological behavior of native cassava starch as well as the factors affecting the rheological behavior of cassava starch. Rheological characterization using rheological models as well as sensitivity analysis of these models were also examined. The model specification of this project was limited to models that relate shear stress to shear rates.

Rheological properties measurement of materials must be subjected to a precised, controlled and quantifiable strain over a given time and the material parameters such as modulus, hardness, viscosity, stiffness, strength or toughness are determined by considering the subsequent forces [1], [2]. Rheological measurements show how materials react under defined conditions- its performance during practical processing such as mixing, sheeting, binding, baking and proofing [3], [4], [5], [6], [7], [8]. In recent times, the utilization of starch has grown from mere domestic use to highly intensive industrial use. It is used either in its native form or after chemical or physical modifications. Starch is not only a basic food in the human or animal diet; it is also broadly used as raw material in the food industry as well as textile, paper and other industries. Starch is mostly in granular form and has different shapes and sizes depending on its botanical source [9]. Starch is a glucose polymer comprising macromolecules of amylopectin and amylose [10], [11]. Texture is an essential factor in consumers’ perception of food quality and has been studied for several years. Rheological profiling offers an unparalleled insight into the textural, handling, stability and appearance characteristics of starch-based products. Starch functions by building structures in a formulation or recipe. It is the presence of such structures that imparts texture, handling, suspending and appearance attributes to a formulation. The importance of rheological models in the characterization of food behavior cannot be over emphasized. Rheological models are used, together with experimental data, to estimate values of parameters that help characterize the rheological behavior of a food samples. One such model is that of Herschel Bulkley model which has been used extensively to characterize foods that exhibit yield stress. Rheological models sometimes called Flow models, this can also be used to derive expressions for volumetric flow rates and velocity profiles in tube and channel flows, and in the analysis of heat transfer phenomenon. Quite a number of these models can be encountered in rheology literature [12]. The applied force is essential for letting the fluid to flow because of fluid friction and this friction has to be overcome before the fluid can flow. Rheological models give a surmised description of fluids by communicating the mathematical relationship between shear stress and shear rates [13]. Mathematical model is regarded as a decision tool that assists decision makers in effectively dealing with complex issues such as rheology and oil spillage on soil surfaces. Such information can be key in decision-making for further experiments and can enable the development of robust and reliable protocols for chemical synthesis, analytical methods or biological assays [14].

There are different models used to measure rheological properties. This research work used models such as power law model, Herschel-Bulkley model, Bingham Plastic model, Prandtl-Eyring model and Robertson-Stiff model. This work did not explain all the available testing methods and general reviews of rheology [15], [16], [17]. A lot of work has been done on rheological testing of foods [12], [18], [19], [20], [21] and cereal products [22], [23], [24], [25].

### 2 Experimental design, materials and methods

Cassava starch tubers were purchased from the local market.

#### 2.1 Flow properties measurement

The sample solution was prepared by dissolving the required quantity based on the required composition needed. 20.00, 30.00, 40.00, 50.00 g of the cassava starch powder was carefully weighed with the aid of a weighing balance and was dissolved in a 400 ml of clean warm water inside a 600 ml beaker until a solution was formed so as to make a reconstituted product. The sample was transferred into the temperature controlled water bath in order to form an aqueous gel which was later placed in a cold water bath (4 °C) medium so as to facilitate the drop in the temperature of the gel to 70, 60, 50, 40 and finally 30 °C. The Ofite viscometer was used in determining the flow characteristics in terms of shear rate and shear stress. A bob of radius 1.8415 cm was used at speeds of 3, 6, 30, 60, 100, 200, 300 and 600 rpm to effectively determine the dial deflection so as to evaluate the shear stress and shear strain.

#### 2.2 Statistical evaluation of rheological models

The least square method was used in evaluating model parameters for each model based on the data obtained from the rheological experiment. This method was chosen due to the following assumptions:

I. The scatter follows a Normal distribution
II. Errors are random errors that are independent and identically distributed with mean of zero and variance, *σ*^2^.

Considering *P* number of data points (*τ_i_*,*γi*), least-square is expressed mathematically in Eq. (1) below.

(1) $$RSS\;(µ)=\sum_{i=1}^{P}{\left(\tau i-f(\gamma i,µ)\right)}^{2}=\in^{2}$$

*RSS* (*µ*) representing the residual sum of squares.
∈ represents random errors.

*µ* representing the value(s) of model parameters that gives minimum *RSS* (also called Least-Square estimators). *µ* has to be determined such that *RSS* (*µ*) will be minimum. Therefore, for the sum of squares to be minimum the partial differential $\frac{\delta \mathrm{RSS}(µ)}{\delta µ}$ **= 0.** The experimental data were fitted to the models using the method above on MATLAB 8.0 to obtain model curve-fits, their corresponding model parameters, residual plots, RMS and RSS values which are necessary for model optimization.

#### 2.3 Preparation of starch from the cassava roots

The cassava tubers purchased were peeled and washed thoroughly. After which the roots were crushed and grinded in the market using a local grinder. The grinded cassava was then soaked in water and screened by passing it through a screening bag to remove the shafts and other unnecessary products. The filtrate was then allowed to settle for a period of one and a half days, after which the solution was dewatered by a simple process of decantation. The resulting product was starch of a moisture content of 36.5%.

#### 2.4 Determination of moisture content of starch

The moisture content was evaluated using [26], [27], [28]. 2.00 g of the starch was weighed into two different empty Petri dishes. The dishes were then placed into an oven at 150 °C for 4 h. The dried starch was immediately transferred into a desiccator until it cooled, and it was then weighed. The weight-loss expressed as a percentage was taken as the percent moisture. The result was obtained as the average of the two independent determinations from both samples.

(2) $$\%\mathrm{Moisture}=\frac{W1-W2}{W3}\times 100\%$$

where:

*W*1= weight of sample ± Petri dish before drying (g)
*W*2 = Weight of sample ± Petri dish after drying (g)
*W*3 = Weight of sample (g)

### 3 Rheological experiment

20.00, 30.00, 40.00, 50.00 g of the cassava starch was carefully weighed with the aid of a weighing balance and was dissolved in a 400 ml of clean warm water inside a 600 ml beaker until a solution was formed so as to make a reconstituted product. The sample was transferred into the temperature controlled water bath in order to form an aqueous gel which was later preferably placed in a cold water bath (4 °C) medium so as to facilitate the drop in the temperature of the gel to 70, 60, 50, 40 and finally 30 °C. The Ofite viscometer was used in determining the flow characteristics in terms of shear rate and shear stress. A bob of radius 1.8415 cm was used at speeds of 3, 6, 30, 60, 100, 200, 300 and 600 rpm to effectively determine the dial deflection so as to evaluate the shear stress and shear strain.

#### 3.1 Determination of gelatinization temperature

A thermometer was inserted into the beaker before placing it into the water bath. The solution was stirred continuously until its colour became milky and thickened. This is the gel point and the temperature at this point was read off as the gelatinization temperature. This was done for each starch concentration.

### 4 Rheological model optimization

Residual mean squares, Residual sum of squares and Coefficient of determination used as statistical tools to evaluate the error variance for each model.

(i) $\mathrm{RMS}=\frac{\mathrm{RSS}}{\mathrm{Degree of freedom}}=\frac{\mathrm{RSS}}{N-M}$

*RMS* representing the residual mean squares
*N* representing the number of data
*M* representing the number of parameters in a model

(ii) $R-\mathrm{squared}=1-\frac{\mathrm{RSS}}{\mathrm{TSS}}$

RSS representing the Residual sum of squares.
TSS representing the Total sum of squares.

(iii) $\mathrm{Residuals}=\tau -\tau^{\prime }$

*τ* representing the observed values.
$\tau^{\prime }$ representing the predicted values.

The residual plot is a graph showing the residuals vs the independent variable (γ).

#### 4.1 Model curve-fits and their corresponding residual plot analysis

##### 4.1.1 Bingham plastic (*τ* = *τ*_o_ + *μ*_p_*γ*)

**Fig. 1:**
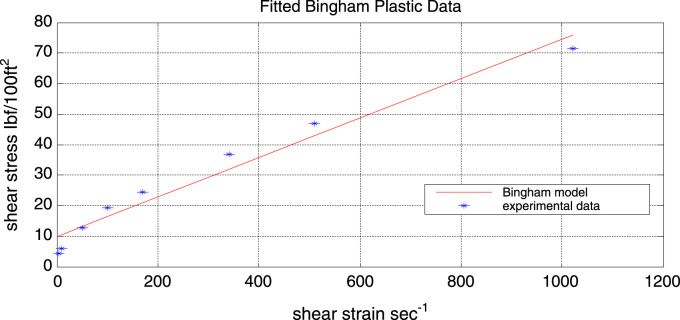
Experimental data and fitted Bingham plastic flow curve.

**Fig. 2:**
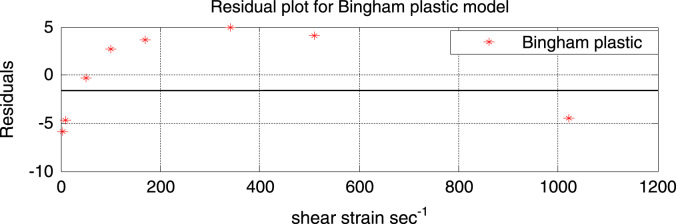
Residual plot of the Bingham plastic model.

Experimental data was fitted into the Bingham plastic model and the corresponding model parameters were evaluated. Fig. 1 represents the fitted model while Fig. 2 represents the corresponding residual plot. The model parameters *τ*_o_ and *μ*_p_ were found to be

*τ*_o_ = 9.8566 Ibf/100 ft^2^ (Yield stress)
*μ*_p_ = 0.0647 Ibf s/100 ft^2^ (Plastic viscosity)

From the above plot it can be clearly seen that the Bingham plastic model gives a very poor fit to the experimental data.

Observations from the residual plot show that the residual do not follow a random distribution or pattern and almost all the residuals are far away from the reference line (residual = 0) indicating a poor fit. Residuals between shear rate of 102.18 s^−1^ to 510.9 s^−1^ lie above the reference line (residual = 0), that is there are positive residuals while the rest of the residual point lie below the reference line indicating negative residuals.

##### 4.1.2 Power-law model (*τ* = *K* γ *^n^*)

**Fig. 3:**
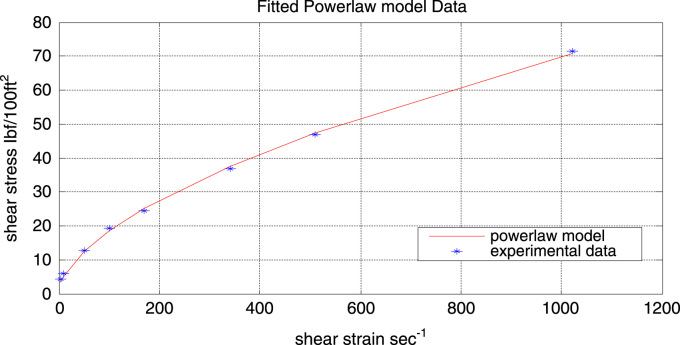
Experimental data and fitted Power-law flow curve.

The power law model gave a better fit than the Bingham plastic model as illustrated in Fig. 3, which shows the fitted power-law model. The model parameter *K*, **γ**, *n* were evaluated and found to be:

*K* = 1.2831 lbf s/100 ft^2^ (Consistency index) which depicts the thickness of the fluid.

*n* = 0.5789 (Flow index) indicates that the fluid is Pseudo-plastic i.e. *n*< 1

**Fig. 4:**
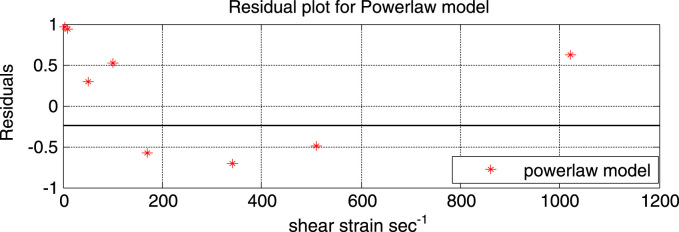
Residual plot of the Bingham plastic model.

Fig. 4 illustrates the residual plot of the power-law model. It can be observed from the plot that the scatter follow a random distribution along the entire range of shear rates. It can also be seen that compared to the Bingham plastic model the residual points of the power-law model are closer to the reference line (residual = 0). Both points indicating that the model gives a good fit.

##### 4.1.3 Herschel Bulkley model (*τ* = *τ*_o_ + *K* γ*^n^*)

**Fig. 5:**
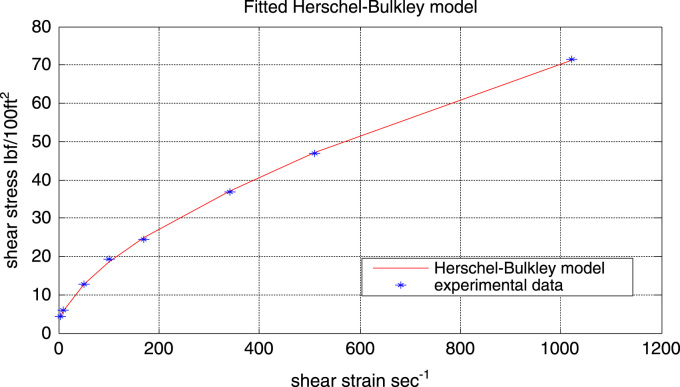
Experimental data and fitted Herschel-Bulkley flow curve.

The Herschel-Bulkley model which is just a modification of the power-law model by the inclusion of the yield stress (*τ*_o_). From Fig. 5, it can be observed that the Herschel-Bulkley model gave a very good fit with model parameters:

τ_o =_ 1.6825Ibf/100 ft^2^ (Yield stress)
K= 0.9893Ibf.s/100 ft^2^ (Consistency index)
n = 0.6138 (flow index)

**Fig. 6:**
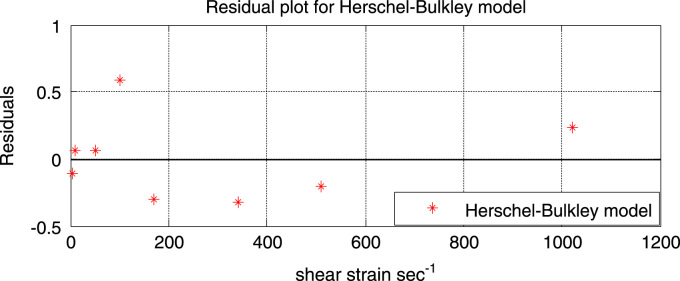
Residual plot of the Herschel-Bulkley model.

Although the fitted flow curves of both the power-law and the Herschel-Bulkley look alike, dissimilarities can be seen in their residual plots (see Fig. 6).

**Fig. 7:**
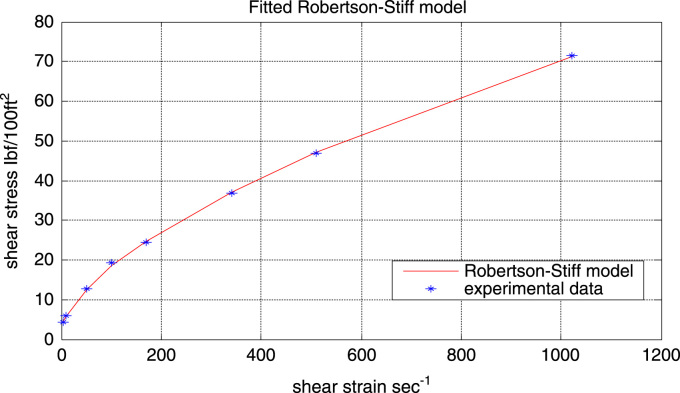
Experimental data and fitted Robertson-Stiff flow curve.

Fig. 6 shows the residual plot of the Herschel-Bulkley model. It can be ascertained from the plot that the scatter follow a random distribution along the entire range of shear rates. It can also be seen that compared to the Bingham plastic model and the power-law model, the residual points of the Herschel-Bulkley model are closer to the reference line (residual = 0). Also since the Herschel-Bulkley model gave better predictions than the power-law model throughout the entire range of shear rate (especially at higher shear rates), the Herschel-Bulkley can therefore be depended upon to give accurate predictions at higher shear rates outside the range used in this project (Fig. 7).

##### 4.1.4 Robertson-Stiff model (*τ* = *A* (*γ*_o_+ *γ*)*^B^*)

Robertson-Stiff model is quite different from the other models being the only model with the yield shear rate (*γ*_o_). The model parameters were evaluated to be:

*A* = 1.1314 Ibf s^0.4026^/100 ft^2^ (the unit of *A* depends on the value of *B*)
*B* = 0.5974
*γ*_o_ = 5.1561 s^-1^

**Fig. 8:**
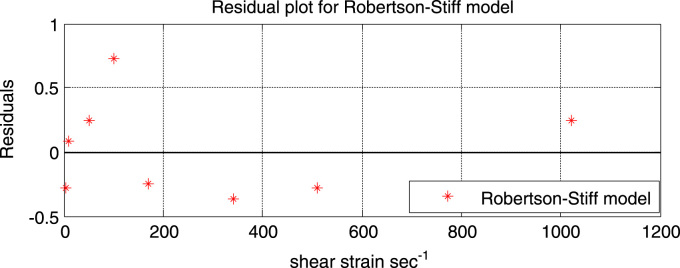
Residual plot of the Robertson-Stiff model.

Although the fitted flow curves for Robertson-Stiff, power-law and the Herschel-Bulkley model all look similar, dissimilarities can be seen in their residual plots (see Fig. 8).

From Fig. 8 it can be seen that the scatter of the residual plot follows a random distribution along the entire range of shear rates. The residual points are also close to the reference line (residual = 0). The model also gives a good fit.

##### 4.1.5 Prandtl-Eyring model (*τ* = Asinh^-1^(γ/B)

**Fig. 9:**
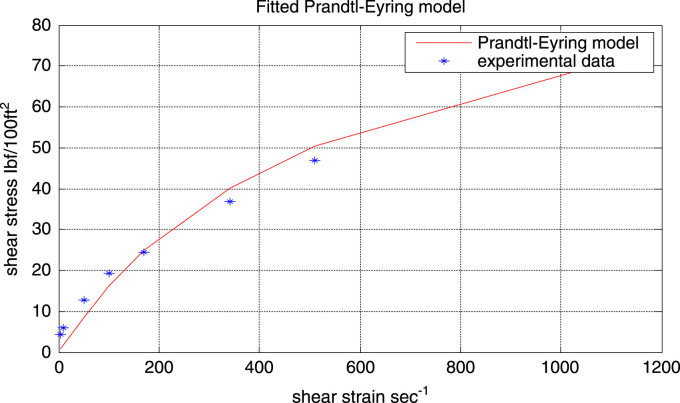
Experimental data and fitted Prandtl-Eyring flow curve.

Fig. 9 represents the fitted flow curve for the Prandtl-Eyring model. The model parameter *A* and *B* where estimated thus:

*A* = 26.7032
*B* = 159.0655

Although it can be observed from the fitted flow curve that the model does not fit the data accurately, this model however fits better than the Bingham plastic model.

**Fig. 10:**
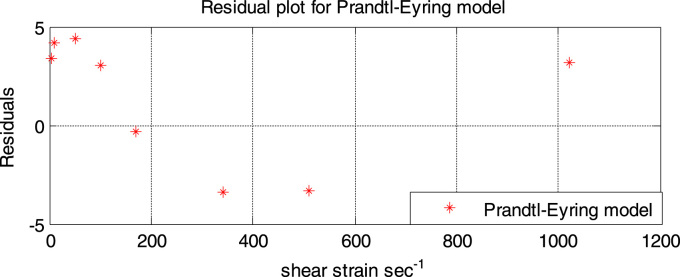
Residual plot of the Prandtl-Eyring model.

Fig. 10 shows the residual plot for the Prandtl-Eyring model. From this plot it can be seen that the scatter are not randomly distributed, and the residual points are far away from the reference line (residual = 0). This suggests that the model does not accurately give a good fit. However in comparison with the Bingham plastic model, the residual points of the Prandtl-Eyring model are closer to the reference line (residual = 0). This indicates that the Prandtl-Eyring model gives a much better fit than the Bingham plastic model.

### 5 Sensitivity analysis

Sensitivity analysis was carried out on each model using the multiple-factor-at-a-time approach (MFAT). The influence of state variables like concentration and temperature on the accuracy of the Bingham Plastic model was examined. Fig. 14 shows the result when the sensitivity analysis at constant concentration and varied temperature was carried out on the Bingham plastic model (Table 1, Table 2, Table 3, Table 4, Table 5, Table 6, Table 7, Table 8, Table 9, Table 10, Table 11).

**Table 1:** Result summary of rheological optimization using least-square method at 700 C and 50 g/L.

Model	*RSS* Ibf^2^/100ft^4^	*RMS* Ibf^2^/100ft^4^	*R*^2^	Evaluated parameters
Bingham Plastic	138.2484	23.0414	0.9626	*τ*_o_ = 9.8566 Ibf/100 ft^2^
				*μ*_p_ = 0.0647 Ibf.s/100 ft^2^
Power-law	3.6559	0.6093	0.999	*K* = 1.2831 Ibf s/100 ft^2^
				*n* = 0.5789
Herschel-Bulkley	0.661	0.1322	0.9998	*τ*o = 1.6825 Ibf/100 ft^2^
				*K* = 0.9893 Ibf s/100 ft^2^
				*n* = 0.6138
Robertson-Stiff	1.0001	0.2	0.9997	*A* = 1.1314 Ibf s^0.4026^/100 ft^2^
				*B* = 0.5974
				*γ*o = 5.1561 s^-1^
Prandtl-Eyring	89.9357	14.9893	0.9757	*A* = 26.7032
				*B* = 159.0655

**Table 2:** Sensitivity analysis of the Bingham plastic model at constant concentration.

	Bingham plastic model			50 g/L concentration
Temperature °C	*RSS* Ibf^2^/100ft^4^	*RMS* Ibf^2^/100ft^4^	*R*^2^	Evaluated parameters
70	138.2484	23.0414	0.9626	*τ*_o_ = 9.8566 Ibf/100 ft^2^
				*μ*_p_ = 0.0647 Ibf s/100 ft^2^
60	181.5753	30.26255	0.9596	*τ*_o_ = 9.8566 Ibf/100 ft^2^
				*μ*_p_ = 0.0647 Ibf s/100 ft^2^
50	253.0253	42.17088333	0.9555	*τ*_o_ = 9.8566 Ibf/100 ft^2^
				*μ*_p_ = 0.0647 Ibf s/100 ft^2^
40	264.9318	44.1553	0.9621	*τ*_o_ = 9.8566 Ibf/100 ft^2^
				*μ*_p_ = 0.0647 Ibf s/100 ft^2^
30	287.4594	47.9099	0.9672	*τ*_o_ = 9.8566 Ibf/100 ft^2^
				*μ*_p_ = 0.0647 Ibf s/100 ft^2^

**Table 3:** Sensitivity analysis of the Bingham plastic model at constant temperature.

	Bingham plastic model			70 °C Temperature
Concentration g/L	*RSS* Ibf^2^/100ft^4^	*RMS* Ibf^2^/100ft^4^	*R*^2^	Evaluated parameters
50	138.2484	23.0414	0.9626	*τ*_o_ = 9.8566 Ibf/100 ft^2^
				*μ*_p_ = 0.0647 Ibf s/100 ft^2^
75	302.7926	50.4654	0.9618	*τ*_o_ = 15.3025 Ibf/100 ft^2^
				*μ*_p_ = 0.0947 Ibf s/100 ft^2^
100	720.1774	120.0296	0.9672	*τ*_o_ = 20.8591 Ibf/100 ft^2^
				*μ*_p_ = 0.1580 Ibf s/100 ft^2^
125	1493.3	248.8857	0.9541	*τ*_o_ = 29.9058 Ibf/100 ft^2^
				*μ*_p_ = 0.1909 Ibf s/100 ft^2^

**Table 4:** Sensitivity analysis of the power-law model at constant concentration.

	Power-law model			50 g/L concentration
Temperature °C	*RSS* Ibf^2^/100ft^4^	*RMS* Ibf^2^/100ft^4^	*R*^2^	Evaluated parameters
70	3.6559	0.6093	0.999	*K* = 1.2831 Ibf s/100 ft^2^
				*n* = 0.5789
60	5.7666	0.9611	0.987	*K* = 1.5111 Ibf s/100 ft^2^
				*n* = 0.5697
50	3.1803	0.5301	0.9994	*K* = 1.7794 Ibf s/100 ft^2^
				*n* = 0.5629
40	7.415	1.2358	0.9989	*K* = 1.7685 Ibf s/100 ft^2^
				*n* = 0.5786
30	5.3505	0.8917	0.9994	*K* = 1.6044 s/100 ft^2^
				*n* = 0.6076

**Table 5:** Sensitivity analysis of the power-law model at constant temperature.

	Power-law model			70 °C Temperature
Concentration g/L	*RSS* Ibf^2^/100ft^4^	*RMS* Ibf^2^/100ft^4^	*R*^2^	Evaluated parameters
50	3.6559	0.6093	0.999	*K* = 1.2831 Ibf s/100 ft^2^
				*n* = 0.5789
75	12.4135	2.0689	0.9984	*K* = 2.0729 Ibf s/100 ft^2^
				*n* = 0.5655
100	12.7359	2.1226	0.9994	*K* = 2.4644 Ibf s/100 ft^2^
				*n* = 0.6119
125	24.255	4.0425	0.9993	*K* = 4.0425 Ibf s/100 ft^2^
				*n* = 0.5689

**Table 6:** Sensitivity analysis of the Herschel-Bulkley model at constant concentration.

	Herschel-Bulkley model			50 g/L concentration
Temperature °C	*RSS* Ibf^2^/100ft^4^	*RMS* Ibf^2^/100ft^4^	*R*^2^	Evaluated parameters
70	0.661	0.1322	0.9998	*τ*_o_ = 1.6825 Ibf/100 ft^2^
				*K* = 0.9893 Ibf s/100 ft^2^
				*n* = 0.6138
60	2.2883	0.4577	0.9995	*τ*_o_= 2.0336 Ibf/100 ft^2^
				*K* = 1.1324 Ibf s/100 ft^2^
				*n* = 0.6085
50	1.0119	0.2024	0.9998	*τ*_o_ = 1.6396 Ibf/100 ft^2^
				*K* = 1.4561 Ibf s/100 ft^2^
				*n* = 0.5897
40	2.0358	0.4072	0.9997	*τ*_o_ = 2.0358 Ibf/100 ft^2^
				*K* = 1.3242 Ibf s/100 ft^2^
				*n* = 0.6175
30	0.6807	0.1361	0.9999	*τ*_o_ = 2.2074 Ibf/100 ft^2^
				*K* = 1.2596 Ibf s/100 ft^2^
				*n* = 0.6403

**Table 7:** Sensitivity analysis of the Herschel-Bulkley model at constant temperature.

	Herschel-Bulkley model			70 °C Temperature
Concentration g/L	*RSS* Ibf^2^/100ft^4^	*RMS* Ibf^2^/100ft^4^	*R*^2^	Evaluated parameters
50	0.661	0.1322	0.9998	*τ*_o_ = 1.6825 Ibf/100 ft^2^
				*K* = 0.9893 Ibf s/100 ft^2^
				*n* = 0.6138
75	3.0026	0.6005	0.9996	*τ*_o_ = 3.3595 Ibf/100 ft^2^
				*K* = 1.4439 Ibf s/100 ft^2^
				*n* = 0.6142
100	2.9597	0.5919	0.9999	*τ*_o_ = 3.1689 Ibf/100 ft^2^
				*K* = 1.9776 Ibf s/100 ft^2^
				*n* = 0.6416
125	2.0358	0.4072	0.9997	*τ*_o_ = 3.1894 Ibf/100 ft^2^
				*K* = 3.4542 Ibf s/100 ft^2^
				*n* = 0.5908

**Table 8:** Sensitivity analysis of the Robertson-Stiff model at constant concentration.

	Robertson stiff model			50 g/L concentration
Temperature °C	*RSS* Ibf^2^/100ft^4^	*RMS* Ibf^2^/100ft^4^	*R*^2^	Evaluated parameters
70	1.0001	0.2	0.9997	*A* = 1.1314 Ibf s^0.4026^/100 ft^2^
				*B* = 0.5974
				*γ*_o_ = 5.1561 s^-1^
60	2.4003	0.4801	0.9995	*A* = 1.3364 Ibf s^0.4026^/100 ft^2^
				*B* = 0.5877
				*γ*_o_ = 5.02071 s^-1^
50	1.2291	0.2458	0.9998	*A* = 1.6428 Ibf s^0.4026^/100 ft^2^
				*B* = 0.5746
				γo= 3.1561 s^-1^
40	2.9577	0.5915	0.9996	*A* = 1.5688 Ibf s^0.4026^/100 ft^2^
				*B* = 0.5962
				*γ*_o_ = 4.8712 s^-1^
30	1.2911	0.2582	0.9999	*A* = 1.4448 Ibf s^0.4026^/100 ft^2^
				*B* = 0.6230
				*γ*_o_ = 4.3726 s^-1^

**Table 9:** Sensitivity analysis of the Robertson-Stiff model at constant temperature.

	Robertson stiff model			70 °C Temperature
Concentration g/L	*RSS* Ibf^2^/100ft^4^	*RMS* Ibf^2^/100ft^4^	*R*^2^	Evaluated parameters
50	1.0001	0.2	0.9997	*A* = 1.1314 Ibf s^0.4026^/100 ft^2^
				*B* = 0.5974
				*γ*_o_ = 5.1561 s^-1^
75	4.9243	0.9849	0.9994	*A* = 1.7902 Ibf s^0.4026^/100 ft^2^
				*B* = 0.5871
				*γ*_o_ = 5.9465 s^-1^
100	3.9647	0.7929	0.9998	*A* = 2.2377 Ibf s^0.4026^/100 ft^2^
				*B* = 0.6260
				*γ*_o_ = 4.0409 s^-1^
125	13.9958	2.7992	0.9996	*A* = 3.7744 Ibf s^0.4026^/100 ft^2^
				*B* = 0.5799
				*γ*_o_ = 3.0094 s^-1^

**Table 10:** Sensitivity analysis of the Prandtl-Eyring model at constant concentration.

	Prandtl-Eyring model			50 g/L concentration
Temperature °C	*RSS* Ibf^2^/100ft^4^	*RMS* Ibf^2^/100ft^4^	*R*^2^	Evaluated parameters
70	89.9357	14.9893	0.9757	*A* = 26.7032
				*B* = 191.6690
60	110.5825	18.4304	0.9754	*A* = 28.5214
				*B* = 146.8925
50	130.4108	21.7351	0.9771	*A* = 31.3211
				*B* = 138.9497
40	170.0923	28.3487	0.9757	*A* = 36.7871
				*B* = 159.6589
30	171.7857	28.631	0.9804	*A* = 44.1196
				*B* = 191.6690

**Table 11:** Sensitivity analysis of the Prandtl-Eyring model at constant temperature.

	Prandtl-Eyring model			70 °C Temperature
Temperature °C	*RSS* Ibf^2^/100ft^4^	*RMS* Ibf^2^/100ft^4^	*R*^2^	Evaluated parameters
50	89.9357	14.9893	0.9757	*A* = 26.7032
				*B* = 191.6690
75	230.7298	38.455	0.9709	*A* = 37.9289
				*B* = 145.7596
100	404.8721	67.4787	0.9816	*A* = 70.7152
				*B* = 197.3360
125	613.7895	102.2982	0.9811	*A* = 76.8097
				*B* = 148.2323
