# Supplementary material for: Data on the rheological behavior of cassava starch paste using different models
Source: Data Brief. 2018 Jun 30;19:2163–77. doi: 10.1016/j.dib.2018.06.112 (PMC6141798; doi:10.1016/j.dib.2018.06.112)
Supplement: Supplementary file 2 — Supplementary material [file mmc2.pdf]

1. [illegible] M.E
2. [illegible]
3. [illegible]
4. [illegible]
